# The First Neocentric, Discontinuous, and Complex Small Supernumerary Marker Chromosome Composed of 7 Euchromatic Blocks Derived from 5 Different Chromosomes

**DOI:** 10.3390/biomedicines10051102

**Published:** 2022-05-10

**Authors:** André Weber, Thomas Liehr, Ahmed Al-Rikabi, Simal Bilgen, Uwe Heinrich, Jenny Schiller, Markus Stumm

**Affiliations:** 1 Medicover Genetics, MVZ Humangenetik Berlin-Lichtenberg, 13053 Berlin, Germany; andre.weber@medicover-genetics.de (A.W.); markus.stumm@medicover.de (M.S.); 2 Institute of Human Genetics, Jena University Hospital, Friedrich Schiller University, Am Klinikum 1, 07747 Jena, Germany; ahmed.al-rikabi@hotmail.com; 3 Medicover Bochum MVZ, 44879 Bochum, Germany; simal.bilgen@medicover.de; 4 Medicover Humangenetik Martinsried, MVZ Martinsried, 81667 München, Germany; uwe.heinrich@medizinische-genetik.de (U.H.); jenny.schiller@medizinische-genetik.de (J.S.)

**Keywords:** molecular cytogenetics, optical genome mapping (OGM), small supernumerary marker chromosomes (sSMC), complex sSMC, discontinuous sSMC, neocentric sSMC, chromothripsis

## Abstract

Background: The majority of small supernumerary marker chromosomes (sSMCs) are derived from one single chromosome. Complex sSMCs instead consist of two to three genomic segments, originating from different chromosomes. Additionally, discontinuous sSMCs have been seen; however, all of them are derived from one single chromosome. Here, we reported a 41 year-old patient with infertility, hypothyroidism, rheumatism, and degenerative spine and schizoaffective disorder, being a carrier of a unique, complex, and discontinuous sSMC. Methods: The sSMC was characterized in detail by banding and molecular cytogenetics including fluorescence in situ hybridization (FISH) and array-comparative genomic hybridization (aCGH), as well as by optical genome mapping (OGM). Results: The neocentric sSMC characterized here contained seven portions of five different chromosomes and was present in ~50% of both peripheral blood cells and buccal mucosa cells. aCGH and OGM revealed gains of 8q12.3q12.3, 8q22.3–8q23.1, 9q33.3–9q34.11, 14q21.1–14q21.1, 14q21.1–14q21.2, 15q21.2–15q21.2, and 21q21.1–21q21.1. Furthermore, glass-needle based microdissection and reverse FISH, as well as FISH with locus-specific probes confirmed these results. The exact order of the involved euchromatic blocks could be decoded by OGM. Conclusions: Among the >7000 reported sSMCs in the literature, this is the only such complex, discontinuous, and neocentric marker with a centric minute shape.

## 1. Introduction

Small supernumerary marker chromosomes (sSMCs) can be found in ~3.3 million people worldwide [1]. sSMC carriers have simultaneously a numerical and a structural chromosomal aberration, and ~75% are practically free of symptoms, as ‘their sSMC’ consists only of genetic material without any genes and/or with genes which are not dosage sensitive [2,3]. sSMCs can have three different shapes: (i) inverted duplication (~60%), (ii) ring (~13.5%), and (iii) centric minute shape (~24.5%); as well, there are ~2% of neocentric sSMCs [1]. The centric minute shape group falls into sSMCs derived from one single chromosome, and those derived from two different chromosomes (~50%, each). sSMCs from the latter group are called complex sSMCs [4]. Of around 89% of these cases derived from a balanced translocation of one of the parents (~23% paternal and ~77% maternal derived), ~11% are de novo (Table 1). Only one complex sSMC case was reported to be derived from three different chromosomes: a 47,XX,+der(7)t(X;5;7)(p22.1;q35;p13q21)dn [5].

Furthermore, there have also been so-called discontinuous sSMCs reported [1,6], which all are derived from two to many or an unclear reported number of parts of one single chromosome (Table 2). Yet, 51 such cases are known, and most of them (~80%) consist of 2 to 4 normally not connected parts of a chromosome. Few cases are known with sSMCs consisting of five or more such ‘randomly’ connected chromosomal blocks (Table 2). To explain these highly rearranged derivative chromosomes in an otherwise normal karyotype, it is suggested that these are remains of incomplete trisomic rescue due to chromothripsis [6,7].

Here, we reported the first complex, neocentric, and at the same time discontinuous sSMC derived from five different chromosomes and seven different euchromatic regions. The sSMC was characterized by means of banding and molecular cytogenetics, molecular karyotyping, as well as the new approach of optical genome mapping (OGM).

## 2. Materials and Methods

### 2.1. Case Report

A 41 year-old patient presented to genetic counselling with infertility in connection with already diagnosed hypergonadotropic hypogonadism, hyperhidrosis, small testicles, and gynecomastia. In addition, he reported to suffer from hypothyroidism, rheumatism, and degenerative spine and schizoaffective disorder.

### 2.2. Cytogenetic Analysis

Chromosome preparations were obtained from stimulated lymphocyte cultures (Lymphogrow medium, Cytocell, Cambridge, UK), and GTG banded after semi-automatic harvesting (Hanabi, ADS Biotec) following standard laboratory procedures [8]. The preparations were evaluated using Ikaros software (Metasystems, Altlussheim, Germany). A total of 33 metaphases were analyzed. Furthermore, buccal mucosa was acquired, and studied by interphase fluorescence in situ hybridization (FISH) using locus-specific probes being present on the sSMC (see below).

### 2.3. Array-Comparative Genomic Hybridization (aCGH)

After extraction of genomic DNA from peripheral blood, aCGH was performed by SurePrint G3 Unrestricted HD-CGH Microarray ISCA v2, 4 × 180 K (Agilent Technologies, Santa Clara, CA, USA) according to standard procedures [9]. Data acquisition was performed on an Innoscan 710 scanner (Innopsys), quantified with feature extraction software, and data were analyzed by Agilent CytoGenomics software (Vers. 4.0.3; Agilent Technologies, Santa Clara, CA, USA). The following conventional settings were used: aberration algorithm ADM-2, threshold 6.0, window size 2 kb, filter ≥ 3 probes, Log_2_Ratio ≥ 0.25, and genome build GRCh37/hg19.

### 2.4. Molecular Cytogenetic Analysis

Microdissection was performed according to standard laboratory procedures in which the sSMC could be isolated. The sSMC-specific DNA was then amplified by degenerated oligonucleotide primed polymerase chain reaction (DOP-PCR) and used in reverse FISH as probe [10]. 

Additionally used FISH probes were selected according to aCGH results and hybridized in 3-color FISH experiments as described elsewhere [11]. Following probes were applied, with genome build GRCh37/hg19:

- RP11-188I6 in 8q22.3 (chr8: 104,806,563-104,945,966);
- RP11-395N15 in 9q34.11 (chr9: 130,860,984-131,055,030);
- RP11-99L13 in 14q21.1 (chr14: 44,795,456-44,973,797);
- RP11-416K5 in 15q21.2 (chr15: 50,385,284-50,543,688);
- RP11-802B2 in 15q21.2 (chr15: 50,586,357-50,763,569); and
- RP11-150L16 in 21q21.1 (chr21: 19,155,572-19,345,359).

Five to ten metaphases with sSMC were evaluated per probe or probe set under a Zeiss Axioplan fluorescence microscope (Jena, Germany) with suited filter sets and ISIS software (MetaSystems, Altlussheim, Germany). 

### 2.5. Optical Genome Mapping (OGM)

For ultra-high molecular weight DNA extraction and labeling, a minimum of 650 µL of whole peripheral blood was introduced into the SP Blood & Cell Culture DNA Isolation Kit following manufacturer instructions (Bionano genomics, San Diego, CA, USA—see also [12]). Briefly, after counting, white blood cells were pelleted (2200 g for 2 min) and treated with LBB lysis buffer and proteinase K to release genomic DNA (gDNA). After inactivation of proteinase K by phenylmethylsulfonyl fluoride treatment, genomic DNA was bound to a paramagnetic disk, washed, and eluted in an appropriate buffer. Ultra-high molecular weight DNA was left to homogenize at room temperature overnight. Then, DNA molecules were labeled using the DLS (Direct Label and Stain) DNA Labeling Kit (Bionano genomics, San Diego, CA, USA). We labeled 750 ng of gDNA in the presence of Direct Label Enzyme (DLE-1) and DL-green fluorophores. After clean-up of the excess of DL-green fluorophores and rapid digestion of the remaining DLE-1 enzyme by proteinase K, the DNA backbone was counterstained overnight. For de novo assembly and structural variant calling, 5 µL of labeled gDNA solution at a concentration between 4 and 12 ng/µL was loaded in overall 8.5 µL on Saphyr chip and scanned on the Saphyr instrument (Bionano genomics, San Diego, CA, USA). Saphyr chip was run to reach a minimum yield of 1300 Gbp. The de novo assembly and variant annotation pipeline were executed on Bionano Solve software V3.5., and reporting and direct visualizing of structural variants was performed on Bionano Access V1.5.2. Recommended filtering was used and corresponded to the following confidence values: insertion or deletion = 0, inversion = 0.01, duplications = −1, translocation = 0.01, and CNV = 0.99. 

## 3. Results

**Figure 1:**
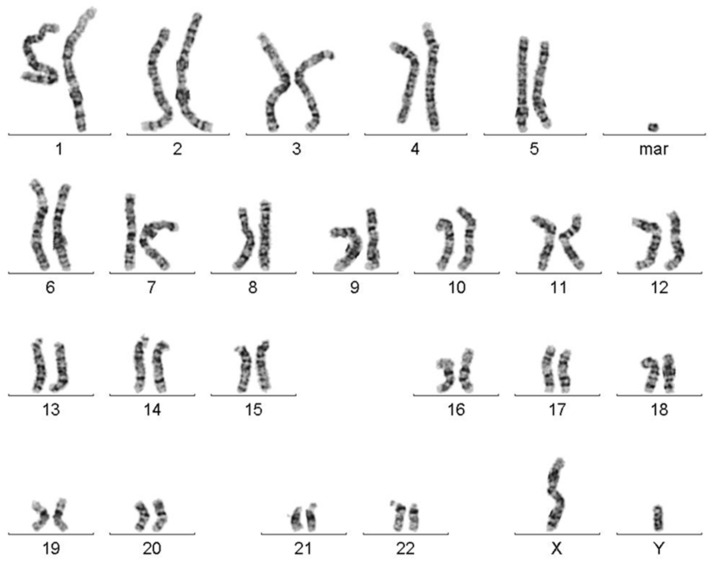
Karyogram after GTG-banding chromosome analysis revealed the presence of a marker chromosome (mar) in ~55% of the analyzed cells.

In stimulated T-lymphocytes from peripheral blood of the index patient, a karyotype mos 47,XY,+mar (18)/46,XY(15) was found (Figure 1).

aCGH analysis provided the unexpected result that the sSMC contained ~15 Mb of euchromatic material derived from 7 different regions distributed on 5 chromosomes and included >220 genes. The average mean log ratio of the detected gains was calculated at ~0.26, which indicated a mosaic constellation of about 50% (Table 3).

**Figure 2:**
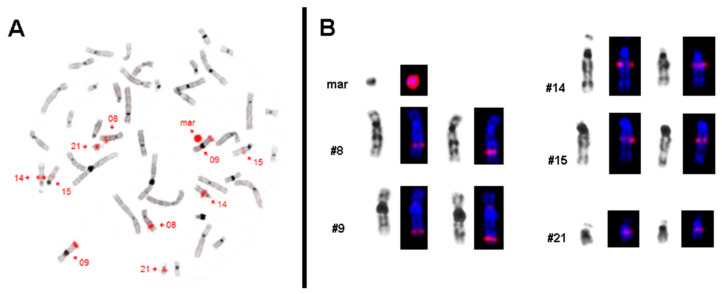
Results of reverse FISH analysis after microdissection of the sSMC. (**A**) The microdissection-derived probe is labeled in red and hybridized back on a metaphase of the patient. The sSMC (mar) is completely stained and the five chromosomes with corresponding signals are highlighted. (**B**) All chromosomes from a patient’s metaphase after hybridizing with microdissection-derived sSMC probe are shown in inverted DAPI (4′,6-diamidino-2-phenylindole) banding as well as stained in DAPI (blue) with the red signals.

These results were confirmed by microdissection and reverse FISH (Figure 2). As in reverse FISH no centromeric region of any chromosome was labeled, the sSMC could be defined as being neocentric. In buccal mucosa, the presence of the sSMC was checked by interphase-FISH with 15q21.2 specific probes RP11-416K5 and RP11-802B2; three signals each were found in 11 of 21 evaluable cells (results not shown), suggesting a similar rate of cells with 47 chromosomes in these epithelial as in peripheral blood cells.

**Figure 3:**
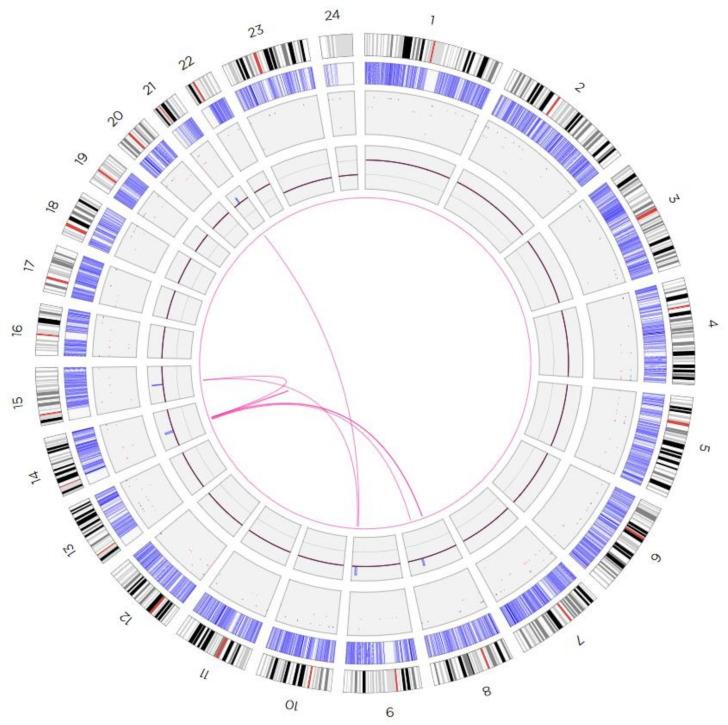
Circos plot depiction of the optical mapping results, showing the interconnections between involved chromosomal regions on the presented sSMC.

**Figure 4:**
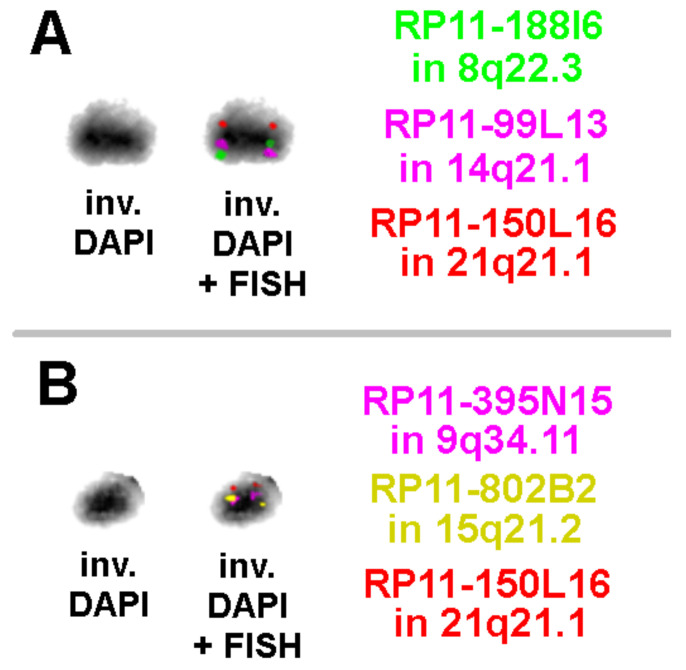
Three-color FISH results on the sSMC with locus-specific probes as specified on the figures are shown; the sSMC is presented as inverted DAPI stained with FISH results. Due to small distance between the probes on the sSMC, the order of the probe signal for 14q21.1 and 8q22.3 (**A**) and those for 9q34.11 and 15q21.2 (**B**) could not be determined by FISH. However, the linear and principal order of them was in concordance with the optical mapping result.

Results of aCGH and reverse FISH were further elucidated by OGM (Figure 3) and metaphase-FISH using overall five locus-specific probes for regions present on the sSMC for 8q22.3, 9q34.11, 14q21.1, 15q21.2, and 21q21.1 (Figure 4).

Summarizing all these results, the most likely composition of this complex sSMC is: der(21q21.1→21q21.1::9q33.3→9q34.1::15q21.2→15q21.2::14q21.1→14q21.1::8q12.3→8q12.3::14q21.2→14q21.2::8q22.3→8q23.1::14q21.1→14q21.1::8q21.1→8q21.1:)

## 4. Discussion

sSMCs are always a problem in clinical diagnostics; in postnatal compared to prenatal settings, sSMC detection is less stressful for the clinician, as there is no decision of life and death connected with the diagnostic outcome. Still, interpretation of the clinical impact of an sSMC is always a topic of interest [13]. However, phenotypic consequences of sSMC presence are dependent on different factors. The main provider of potential clinical problems is the euchromatic imbalance caused by the sSMC [14]. A comparison with previously reported cases can normally help [1]. Moreover, there is an influence of mosaicism for clinical outcomes; it has been shown that even carriers of well-known sSMC-related syndromes may not be physically and/or mentally impaired in the case where the sSMC is only present in a minor part of the body cells [3]. Additionally, uniparental disomy of sSMC’s sister chromosomes can lead to clinical consequences in ~2–5% of the cases [15].

The present case is a real challenge for genetic counselling. The patient reported several signs and symptoms, which might or might not be related to sSMC presence. Male infertility, being one of the patient’s problems, was traced back to the presence of heterochromatic sSMCs as well [16]. However, here, oligoasthenozoospermia or azoospermia with a history of recurrent pregnancy loss in partnership and also small testicles were reported [7]. Whether the additional patient-reported symptoms (hypergonadotropic hypogonadism, hyperhidrosis, gynecomastia, hypothyroidism, rheumatic complaints, degenerative spine disorder, and schizoaffective disorder) are related to the sSMC is difficult to conclude [17]. Still, many of these conditions are also observed in Klinefelter syndrome or are considered to be multifactorially caused; thus, an evaluation to suggest candidate genes for any of the patient’s findings seems not to be indicated here.

sSMCs may carry many genes and nonetheless be non-deleterious for its carrier in the case these genes are not dosage sensitive [14]. In addition, it is a general trend that gain of copy numbers can be better tolerated than loss of copy numbers [18]. Interestingly, there are reports on the so-called directly transmitted unbalanced chromosome abnormalities (UBCAs), being huge gains or losses of chromosomal material (in megabasepair range), which did not cause any or less than expected harm to their carriers [19]. A comparison of the regions gained in the actual case due to sSMC revealed that the three regions 8q22.3q23.1, 14q21.1q21.2, and 21q21.1q21.1 overlapped with such UBCA-regions (Table 4) [20]. Furthermore, a search in the UCSC Genome Browser on Human (GRCh37/hg19) for copy number gains of the affected regions revealed that there are (i) no identical cases reported and (ii) relatively few cases for larger pathogenic gains reported for all regions, apart from 21q21.1q21.1 (Table 4). This could suggest that most if not all regions present on the sSMC contain no or only extremely few dosage-dependent genes, which would agree with the relatively mild symptoms in the case of a 15 Mb gain of euchromatic material. It must also be considered that in both tested tissues (blood and buccal mucosa), the sSMC was present in only 50% of the cells, which also would have a favorable effect on the patient’s health [14].

How such an sSMC could have evolved is difficult to establish; its highly complex and discontinuous structure suggests a formation involving chromothripsis [6]. However, for chromothripsis in the case of a single chromosome origin, discontinuous sSMC, the formation can be attributed to a trisomic rescue event, and at least a rescue of a trisomy of five different chromosomes must be suggested including shattering, loss, and fusion of a few parts. Furthermore, chromothripsis to rescue a triploidy cannot be excluded in the actual case. However, the designation “chromothripsis” only covers the fact that there indeed are only ‘ideas’ of what is behind this mechanism, how it is initiated, regulated, and when it is taking place: involvement of micronuclei and formation of chromatin bridges have been suggested [21,22]. Additionally, the fact that the sSMC is neocentric cannot contribute to solve the question of its formation; yet, reported neocentric sSMCs have either an inverted duplication, ring, or centric minute shape [23]. One discontinuous neocentric case was also reported [24], but no neocentric sSMC derived from different chromosomes are known. Thus, for the actual case, we could only state that some kind of rescue event must have taken place, most likely in the one- to few-cell stage of the embryo, leading to chromosomal shattering and fusion of those segments, which finally formed the reported sSMC.

## 5. Conclusions

Overall, the first case of an obviously chromothripsis-related sSMC composed of seven euchromatic blocks derived from five different chromosomes was reported. Considering that, this is the first such sSMC seen after ~70 years after first case report on sSMCs [25], such cases seem to be the exception than the rule. Still, it cannot be excluded that some of the ~7000 published cases were underestimated in their complexity, due to the lack of corresponding approaches. OGM can be a useful tool to further elucidate such complex structural changes.

## Figures and Tables

**Table 1:** Complex sSMCs by chromosomal origin of the centromeric region.

Chromosomal Origin (Centromere)	Number of Cases			
	Inherited *	De Novo	n.a.	Overall
1	0	0	0	0
2	0	0	0	0
3	0	0	0	0
4	1	0	1	2
5	0	0	0	0
6	0	0	0	0
7	0	1	0	1
8	2	1	0	3
9	6	0	1	7
10	0	1	0	1
11	1	1	0	2
12	1	1	0	2
13	12	0	3	15
13 or 21	6	6	0	12
14	20	4	6	30
14 or 22	0	1	0	1
15	11	5	6	22
16	0	0	0	0
17	0	0	1	1
18	5	1	1	7
19	0	0	1	1
20	0	0	0	0
21	14	0	2	16
22 **	11	2	9	32
X	0	0	0	0
Y	0	0	0	0
**overall**	**90**	**24**	**31**	**155**

* All mat derived—only 21 cases pat; ** excluding der(22)t(11;22)(q23;q11.2) cases.

**Table 2:** Discontinuous sSMCs by chromosomal origin and indicating the number of identified euchromatic blocks.

Chromosomal Origin	Number (#) of Blocks						
	2	3	4	5	>5	# Not Given	Overall
1	1	1	0	1	0	0	**3**
2	1	1	0	0	0	0	**2**
3	1	0	0	0	0	0	**1**
4	1	1	0	0	0	0	**2**
5	1	0	0	0	0	0	**1**
6	0	0	0	0	0	0	**0**
7	0	0	0	0	0	0	**0**
8	3	2	0	1	0	2	**8**
9	0	0	0	0	0	1	**1**
10	0	0	0	0	0	1	**1**
11	1	0	0	0	1	0	**2**
12	0	0	2	0	1	0	**3**
13	1	1	0	0	0	0	**2**
14	0	0	1	0	0	0	**1**
15	3	1	5	0	1	1	**11**
16	0	0	0	0	0	0	**0**
17	1	1	0	0	0	0	**2**
18	1	0	0	0	0	0	**1**
19	1	2	0	1	0	0	**4**
20	1	0	0	0	0	0	**1**
21	2	0	0	0	0	0	**2**
22	1	1	0	0	0	0	**2**
X	1	0	0	0	0	0	**1**
Y	0	0	0	0	0	0	**0**
**overall**	**21**	**11**	**8**	**3**	**3**	**5**	**51**

**Table 3:** Summary of imbalances due to sSMC presence as detected by aCGH.

Chromosome	Type of Imbalance	Size (Mb)	Cytobands and Positions Acc. to GRCh37/hg19
8 *	gain	0.128274	8q12.3q12.3(62474378_62602652)x3[0.5]
8	gain	3.403637	8q22.3q23.1(103083594_106487230)x3[0.5]
9	gain	4.321481	9q33.3q34.11(127319305_131640785)x3[0.5]
14	gain	0.550404	14q21.1(38288122_38838525)x3[0.5]
14	gain	3.819669	14q21.1q21.2(42160061_45979729)x3[0.5]
15	gain	1.009670	15q21.2(49763826_50773495)x3[0.5]
21	gain	1.807907	21q21.1(18282221_20090127)x3[0.5]
overall	gain	15.041042	-

* These data were not available from aCGH but from OMG.

**Table 4:** Comparison of regions present as partial trisomy due to the sSMC in the present patient, UBCA cases [20], and number of pathological cases listed in UCSC in case the corresponding region was duplicated.

Chromosome	Cytobands	UBCAs Reported Acc. to [20]	UCSC—Larger Pathogenic Gain Reported
8	8q12.3q12.3	-	21
8	8q22.3q23.1	dup(8)(q21.2q21.2)	30
9	9q33.3q34.11	-	34
14	14q21.1q21.1	-	10
14	14q21.1q21.2	dup(14)(q13q22)	9
15	15q21.2q21.2	-	5
21	21q21.1q21.1	dup(21)(q11.2~21.1q21.2)	239

## Data Availability

All data are included in this paper.
